# Cancer cells corrupt normal epithelial cells through miR-let-7c-rich small extracellular vesicle-mediated downregulation of p53/PTEN

**DOI:** 10.1038/s41368-022-00192-2

**Published:** 2022-07-19

**Authors:** Weilian Liang, Yang Chen, Hanzhe Liu, Hui Zhao, Tingting Luo, Hokeung Tang, Xiaocheng Zhou, Erhui Jiang, Zhe Shao, Ke Liu, Zhengjun Shang

**Affiliations:** 1 The State Key Laboratory Breeding Base of Basic Science of Stomatology (Hubei-MOST) & Key Laboratory for Oral Biomedicine Ministry of Education, School and Hospital of Stomatology, Wuhan University, Wuhan, China; 2 Shenzhen PKU-HKUST Medical Center (Peking University Shenzhen Hospital), Shenzhen, China; 3 Department of Oral and Maxillofacial Surgery, School and Hospital of Stomatology, Wuhan University, Wuhan, China; 4 Department of Oral and Maxillofacial-Head and Neck oncology, School & Hospital of Stomatology, Wuhan University, Wuhan, China

**Keywords:** Cancer microenvironment, Oral cancer

## Abstract

Tumor volume increases continuously in the advanced stage, and aside from the self-renewal of tumor cells, whether the oncogenic transformation of surrounding normal cells is involved in this process is currently unclear. Here, we show that oral squamous cell carcinoma (OSCC)-derived small extracellular vesicles (sEVs) promote the proliferation, migration, invasion, and epithelial-mesenchymal transition (EMT) of normal epithelial cells but delay their apoptosis. In addition, nuclear-cytoplasmic invaginations and multiple nucleoli are observed in sEV-treated normal cells, both of which are typical characteristics of premalignant lesions of OSCC. Mechanistically, miR-let-7c in OSCC-derived sEVs is transferred to normal epithelial cells, leading to the transcriptional inhibition of p53 and inactivation of the p53/PTEN pathway. In summary, we demonstrate that OSCC-derived sEVs promote the precancerous transformation of normal epithelial cells, in which the miR-let-7c/p53/PTEN pathway plays an important role. Our findings reveal that cancer cells can corrupt normal epithelial cells through sEVs, which provides new insight into the progression of OSCC.

## Introduction

Oral squamous cell carcinoma (OSCC) remains the most common malignant tumor of the lip and oral cavity and the 18^th^ most prevalent neoplasm worldwide in 2020;^1^ it generally begins with epithelial hyperplasia and continues through dysplasia and carcinoma in situ, eventually progressing to invasive carcinoma^2^ (Fig. S1). The pathogenicity of OSCC is associated with genetic, epigenetic, and environmental factors. Unhealthy life habits can lead to gene mutations in normal epithelial cells.^3,4^ The mutated epithelial cells then acquire an enhanced proliferation ability and become immortalized tumor cells, resulting in the formation of OSCC.^5,6^

OSCC is composed of heterogeneous tumor cells and a complicated tumor microenvironment (TME), which contains different noncancerous cells and various extracellular matrix (ECM) components.^7,8^ Cellular communication between cancer cells and noncancerous cells in the TME plays vital roles in cancer progression and therapeutic resistance.^9^ Lately, researchers mainly focused on the interaction between malignant tumor cells and noncancerous cells in the TME, and few examined the communication between cancer cells and normal cells around tumor bulk.^10^ Continuous volume increase is a typical feature of the tumor progressive stage.^11,12^ This increase can be attributed to the proliferation of tumor cells. However, it may also be due to the oncogenic transformation of normal cells surrounding the tumor bulk. Still, whether tumor cells are involved in the precancerous transformation of normal epithelial cells is unclear. Hence, exploring the role of cancer cells in corrupting normal epithelial cells might provide a promising target to treat OSCC.

Cancer-derived extracellular vesicles (EVs), such as small extracellular vesicles (sEVs), are involved in cellular communication by delivering special cargos such as proteins, lipids, and nucleic acids.^13–15^ Our previous studies suggested that melanoma-derived exosomal miR-15-5p can promote proangiogenic switch of cancer-associated fibroblasts (CAFs),^16^ and OSCC-derived microvesicles (MVs) can lead to glycometabolic reprogramming in fibroblasts.^17^ Zomer et al. demonstrated that EVs participate in the phenotypic transmission between malignant tumor cells and less malignant tumor cells.^18^ Given the critical roles of cancer-derived EVs in regulating the TME, exploring the effect of OSCC-derived sEVs on adjacent normal cells is critically important.

In this study, we explored the cellular communication between OSCC cells and normal epithelial cells. We revealed a potential mechanism by which cancer cells corrupt normal epithelial cells. Our studies provide fresh insights into the progression of OSCC.

## Results

### OSCC-derived sEVs enhance the migration and invasion of normal epithelial cells

**Fig. 1:**
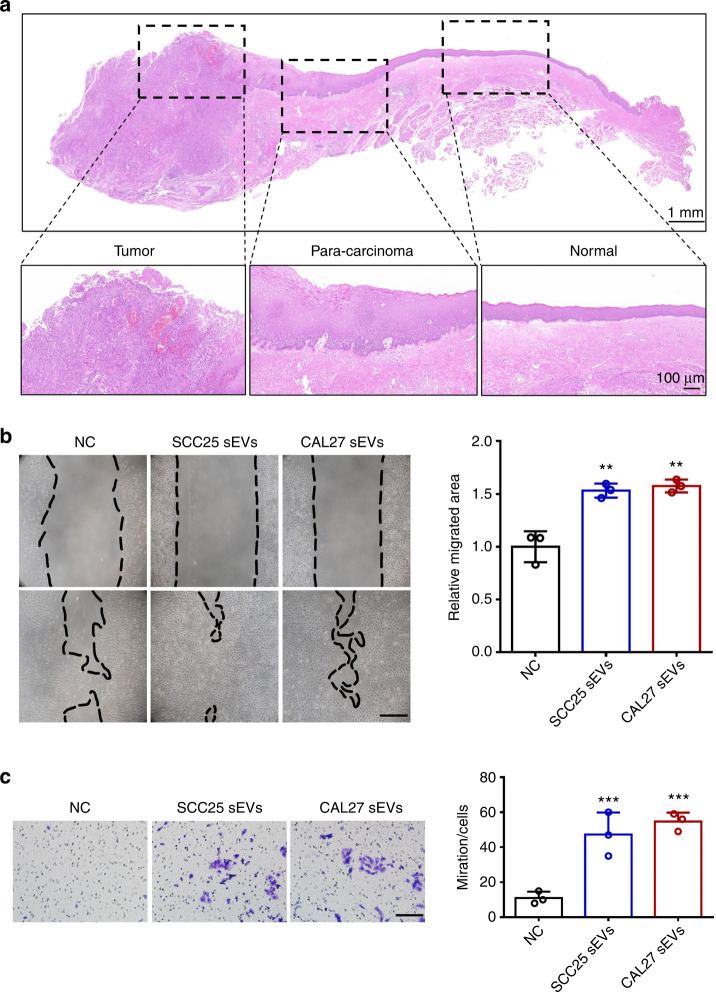
OSCC-derived sEVs promote the migration and invasion of normal epithelial cells. **a** HE staining of OSCC tissues. **b** The migration areas of HIOECs treated with OSCC-derived sEVs. Scale bar, 200 μm. **c** The invasion cells of HIOECs treated with OSCC-derived sEVs. Scale bar, 100 μm

HE staining of OSCC tissues showed atypical hyperplasia between the cancer nest area and the safe surgical margin (Fig. 1a), indicating that cancer cells might induce the precancerous transformation of normal epithelial cells. To verify whether sEVs are involved in this process, differential centrifugation was used to purify the sEVs from the supernatant of OSCC cells as previously described.^19^ To determine whether the obtained nanovesicles were sEVs, we identified them by TEM, dynamic light scattering (DLS) analysis, and western blotting (Fig. S2a–c). The typical cup-shaped morphology, 50–200 nm diameters, and marker protein expression, including calnexin, Hsp90, Tsg101, and CD63, verified that these nanovesicles were indeed sEVs.^19^ The immunofluorescence results indicated that red fluorescence was detected in the cytoplasm of human immortalized oral epithelial cells (HIOECs) (Fig. S2d), indicating that the sEVs were absorbed by normal epithelial cells. During the process of malignant transformation, normal epithelial cells acquire enhanced migration ability and even the ability to infiltrate.^20,21^ We then assessed the migration and invasion capacities of OSCC-derived sEV-treated HIOECs. We found that both SCC25- and CAL27-derived sEVs enhanced the migration ability of HIOECs (Fig. 1b). Moreover, HIOECs acquired the ability to invade after being treated with sEVs, whereas none of the untreated cells broke through the blockade of Matrigel (Fig. 1c). Together, these results demonstrate that OSCC-derived sEVs enhanced the migration ability of normal epithelial cells and endowed them with invasion ability.

### OSCC-derived sEVs enhance proliferation and inhibit apoptosis in normal epithelial cells

**Fig. 2:**
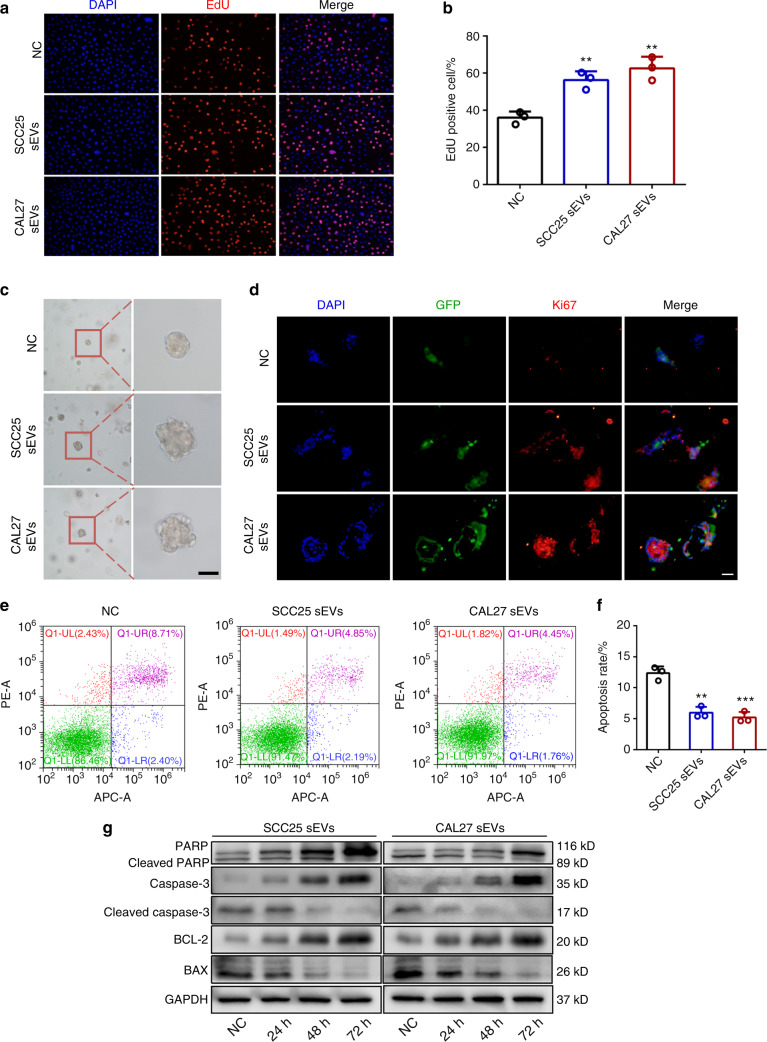
OSCC-derived sEVs enhance the proliferation and inhibit the apoptosis of normal epithelial cells. **a**, **b** The proliferative cells of HIOECs treated with OSCC-derived sEVs. Scale bar, 50 μm. **c** The size and morphology of HIOECs in Matrigel treated with OSCC-derived sEVs. Scale bar, 50 μm. **d** Immunofluorescence staining of Ki67 in HIOECs treated with OSCC-derived sEVs in Matrigel. Scale bars, 100 μm. **e**, **f** The apoptosis rate of HIOECs treated with OSCC-derived sEVs. **g** The expression of apoptosis-related markers in HIOECs treated with OSCC-derived sEVs

Aberrant proliferation and apoptosis are the major characteristics of tumor cells when compared with those of normal cells.^22,23^ Hence, we detected the proliferation ability of HIOECs treated with OSCC-derived sEVs. CCK-8 assay illustrated the proliferation rate increased with the time of sEVs stimulation (Fig. S3a). In addition, the EdU assay showed that the proliferative cells were significantly augmented in the treated groups (Fig. 2a, b). To simulate the real growth environment of cells in the body, we conducted a cell proliferation experiment under 3-dimensional (3D) culture conditions. As shown in Fig. 2c, the spheres in the treated groups were larger and irregular in shape and Ki67 expression increased compared to control group (Fig. 2d). We then determined the apoptosis rate of HIOECs using flow cytometry. We found that the treated cells had a lower apoptosis rate (Fig. 2e, f). Moreover, apoptosis-related markers, including cleaved PARP, cleaved caspase-3, and BCL-2, gradually decreased after stimulation (Fig. 2g). In conclusion, the above results verify that OSCC-derived sEVs can improve the proliferation and inhibit the apoptosis of normal epithelial cells.

### Normal epithelial cells undergo precancerous transformation after treatment with OSCC-derived sEVs

**Fig. 3:**
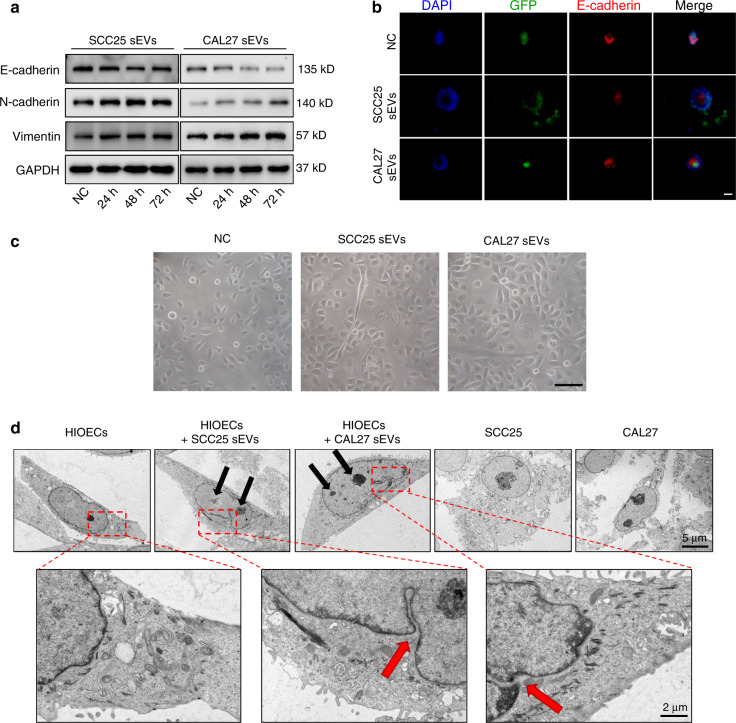
Normal epithelial cells undergo malignant transformation after treatment with OSCC-derived sEVs. **a** The expression of EMT-related markers in HIOECs treated with OSCC-derived sEVs. **b** Immunofluorescence staining of E-cadherin in HIOECs treated with OSCC-derived sEVs in Matrigel. Scale bars, 100 μm. **c** The morphology of HIOECs treated with OSCC-derived sEVs. Scale bar, 50 μm. **d** The ultrastructural features of HIOECs treated with OSCC-derived sEVs. Scale bar, 5 μm

Epithelial-mesenchymal transition (EMT) has crucial roles in the occurrence and development of malignant epithelial tumors.^24,25^ The cell-cell adhesion disappears between epithelial cells during EMT and the cells acquire stronger invasion and transition abilities.^26^ Here, we found EMT-related markers changed significantly under treatment with OSCC-derived sEVs, including the decrease of E-cadherin and the increase of N-cadherin and vimentin (Fig. 3a). The immunofluorescence staining results also confirmed the lower E-cadherin expression in the treated groups (Fig. 3b). Moreover, we found that the morphology of HIOECs transformed from oval to spindle-shaped (Fig. 3c). All of these results verified that OSCC-derived sEVs could enhance the EMT of HIOECs. The precancerous transformation of normal cells is accompanied by ultrastructural changes.^27^ Frequent nuclear-cytoplasmic invaginations and multiple nucleoli were observed in the treated epithelial cells, both of which are typical characteristics of premalignant oral lesions (Fig. 3d). In general, these results demonstrate that normal epithelial cells underwent precancerous transformation when treated with OSCC-derived sEVs.

### The miR-let-7c/p53/PTEN pathway participates in the precancerous transformation of normal epithelial cells

**Fig. 4:**
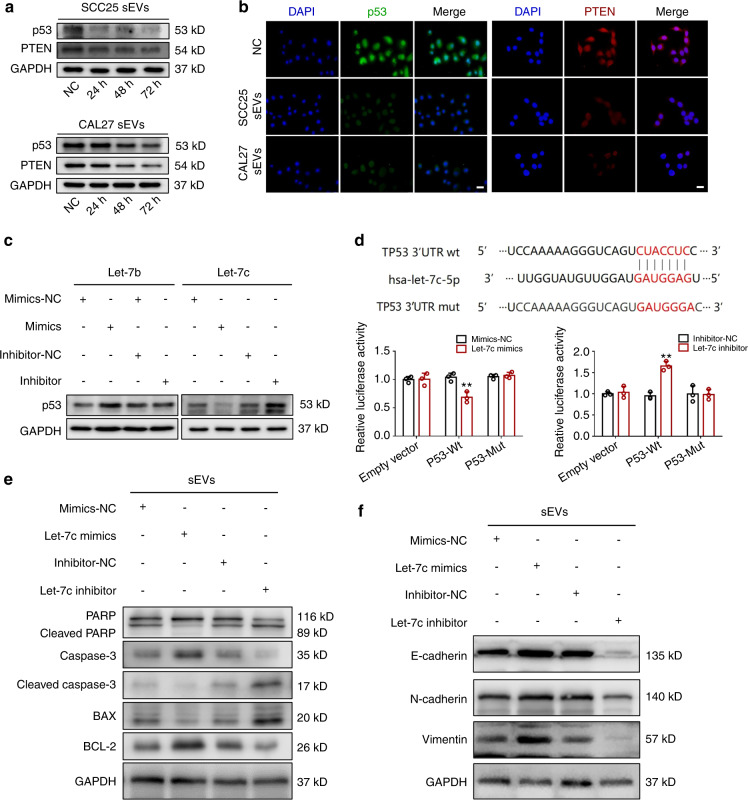
MiRNA let-7c/p53/PTEN pathway participates in the malignant transformation of normal epithelial cells. **a** The expression of p53 and PTEN in HIOECs treated with OSCC-derived sEVs. **b** Immunofluorescence staining of p53 and PTEN in HIOECs treated with OSCC-derived sEVs. Scale bars, 20 μm. **c** The expression of p53 in HIOECs when treated with sEVs derived from OSCC cells that pretreatment with the mimics and inhibitor of let-7b or let-7c. **d** Prediction of binding sites between miR-let-7c and p53, and dual-luciferase activity analysis. **e**, **f** The expression of EMT- and apoptosis-related markers in HIOECs when treated with sEVs derived from OSCC cells that pretreatment with let-7c mimics or let-7c inhibitor

**Fig. 5:**
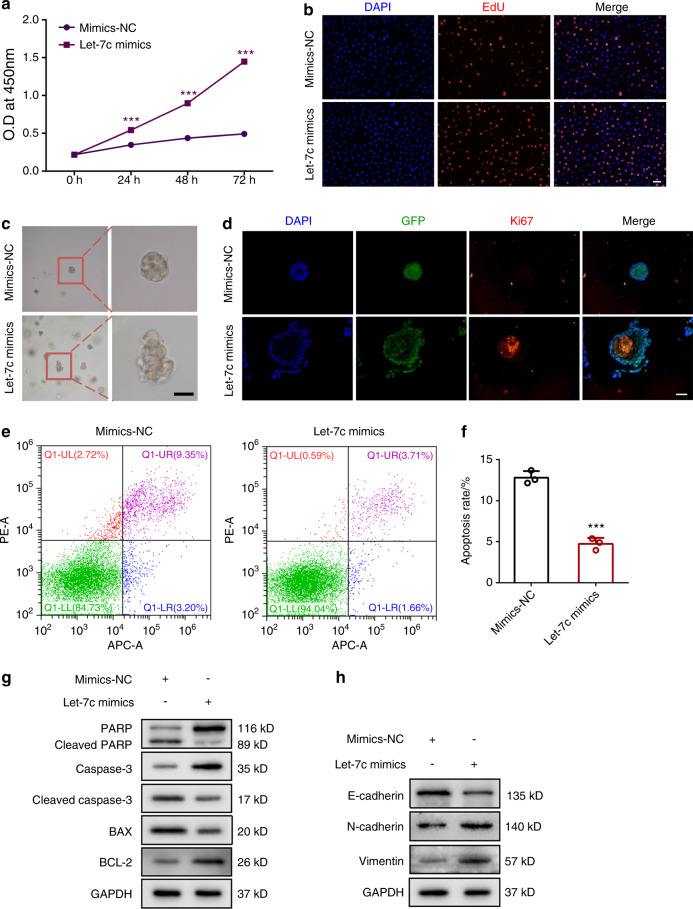
MiRNA let-7c plays a similar role to OSCC-derived sEVs on normal epithelial cells. **a** The proliferation ability of HIOECs transfected with let-7c mimics. **b** The proliferative cells of HIOECs transfected with let-7c mimics. **c** The size and morphology of HIOECs transfected with let-7c mimics in Matrigel. Scale bar, 50 μm. **d** Immunofluorescence staining of Ki67 in HIOECs transfected with let-7c mimics in Matrigel. Scale bars, 100 μm. **e**, **f** The apoptosis rate of HIOECs transfected with let-7c mimics. **g** The expression of apoptosis-related markers in HIOECs transfected with let-7c mimics. **h** The expression of EMT-related markers in HIOECs transfected with let-7c mimics

p53 and PTEN are the most frequently mutated tumor suppressor genes and their mutation is related to the occurrence of various cancers.^28,29^ Previous studies have demonstrated that the mutated genes of OSCC mainly belong to the category of tumor suppressors.^30^ Therefore, we detected the expression of p53 and PTEN in sEV-treated normal cells. As shown in Fig. 4a, b, p53 and PTEN was significantly decreased in the treated cells. sEVs participate in intercellular communication by delivering specific cargos such as proteins, cholesterol, DNA, and miRNA. To predict the miRNAs that target p53, we used four different databases, miRanda, microT, miRmap, and TargetScan for prediction and then took the intersection among the predictions. The results showed that p53 was targeted by ten miRNAs, including miR-let-7a, miR-let-7b, miR-let-7c, miR-let-7d, miR-let-7e, miR-let-7f, miR-let-7g, miR-let-7i, miR-98, and miR-150 (Fig. S3b). In addition, we detected the abundance of these miRNAs by qRT–PCR, and we found that miR-let-7b and miR-let-7c were highly expressed in OSCC-derived sEVs (Fig. S3c). Then cellular miRNAs in HIOECs, CAL27 and SCC25 was detected, and we found that miR-let-7c was substantially expressed in CAL27 and SCC25, whereas miR-let-7b was only abundantly existed in SCC25 (Fig. S3d). The miRNA expression in treated HIOECs confirmed that OSCC-derived miR-let-7c could be effectively transferred to normal epithelial cells (Fig. S3e). Next, mimics and inhibitors of miR-let-7b and miR-let-7c were used to treat HIOECs, and we found that miR-let-7c inhibited the expression of p53 and might play a similar role to OSCC-derived sEVs (Fig. 4c). A dual-luciferase reporter assay revealed miR-let-7c could suppress p53 expression at the transcription level (Fig. 4d). We found that pretreatment with the miR-let-7c inhibitor dampened the ability of tumor sEVs to promote the malignant transformation of HIOECs, whereas pretreatment with miR-let-7c mimics showed the opposite results (Fig. 4e, f). Furthermore, we verified miR-let-7c plays a similar role to OSCC-derived sEVs on normal epithelial cells (Fig. 5 and Fig. S3f–h). Overall, these results verify that the miR-let-7c/p53/PTEN pathway participated in the precancerous transformation of normal epithelial cells.

## Discussion

**Fig. 6:**
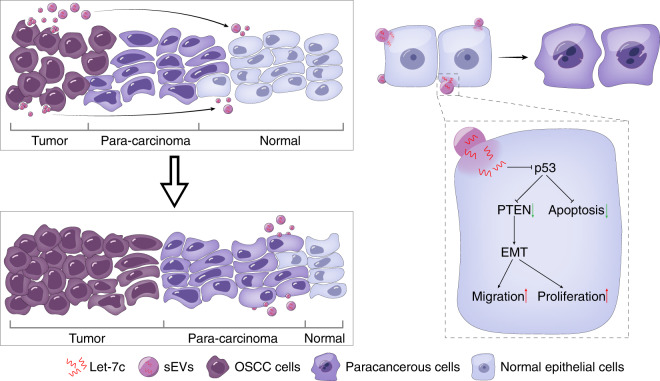
Schematic diagram of the roles of OSCC-derived sEVs in normal epithelial cells. OSCC-derived sEVs promotes the malignant transformation of normal epithelial cells through the miR-let-7c/p53/PTEN pathway

The oncogenic transformation of normal epithelial cells in oral cavity is closely related to genetic, epigenetic, and environmental factors.^3,4^ The long-term stimulation of carcinogenic factors results in the inactivation of tumor suppressors and activation of oncogenes, thus leading to the formation of OSCC.^30^ Today, scholars have made great achievements in detailing tumor-stroma communication.^31,32^ However, few studies have paid attention to the crosstalk between cancer cells and surrounding normal epithelial cells. The volume increase of tumors may be partly due to the malignant transformation of normal cells around tumor mass as well as the self-renewal of cancer cells. Determining whether cancer cells can cause the cancerous alteration of the surrounding normal cells may provide new insight into the development of OSCC in addition to typical mechanism of carcinogenesis. In this study, we explored the cellular communication between OSCC and normal oral epithelial cells. We found that cancer cells can corrupt normal epithelial cells through sEVs exchange. Further study revealed that miR-let-7c is involved in the precancerous alteration of normal epithelial cells (Fig. 6).

EVs, which are secreted by almost all cells, are nanoscale vesicles that are involved in cellular communication by delivering bioactive molecules between different cells. Tumor-derived EVs play important roles in tumor migration, invasion, metastasis, angiogenesis, and immune escape.^33,34^ Our previous studies have found that melanoma-derived exosomes is able to activate the proangiogenic switch of CAFs, thus enhancing tumor angiogenesis.^16^ OSCC-secreted MVs can induce glycobolic reprogramming of fibroblasts, thus promoting tumor progression.^17^ Malgorzata et al. revealed that arginase-1-carrying sEVs can suppress the T-cell response and accelerate tumor progression.^15^ Tumor-derived EVs can produce cancerous characteristics by transporting cancer-promoting molecules to recipient cells.^35^ Given the critical roles of EVs in regulating nontumor cells in the TME, we explored the roles of OSCC-derived sEVs on surrounding normal epithelial cells. We found OSCC-derived sEVs enhance the migration, invasion, proliferation, and EMT of normal epithelial cells but diminish their apoptosis rate. In addition, an increased nuclear area and nuclear-cytoplasmic ratio, frequent nuclear-cytoplasmic invaginations and multiple nucleoli were observed in the sEV-treated epithelial cells. All of these results indicate that normal epithelial cells are corrupted by malignant cancer cells and undergo precancerous transformation, which might partly contribute to the volume increase during the tumor progression stage. Our study further expands the roles of tumor-derived sEVs in switching TME and producing long-distance oncogenic effects.

p53 and PTEN are the most frequently mutated tumor suppressor genes in OSCC.^30^ Sawada et al. found that the frequency of p53 mutations is correlated with the degree of oral epithelial dysplasia, which implies that the acquisition of the mutation is an important step toward OSCC.^36^ In benign cells, p53-related pathways are closely related to the tumorigenesis of OSCC and can regulate cell survival through the transcriptional regulation of PTEN.^37^ Chen et al. found that the phosphorylation and SUMOylation of PTEN were related to the development of oral cancer.^29^ Here, we found that p53 and PTEN were significantly decreased in sEV-treated normal epithelial cells, which further confirmed that malignant transformation occurred in these normal epithelial cells. Previous researches have verified a variety of roles of miRNAs in the carcinogenesis of OSCC.^38^ Here, we found that sEV-derived miR-let-7c can target p53 and negatively regulate its expression in normal epithelial cells. The mimics of miRNA let-7c have a similar effect, whereas the inhibitor of miR-let-7c dampens the changes caused by OSCC-derived sEVs. MiR-let-7c has been shown to be involved in the development of various diseases.^39–41^ Although many studies have demonstrated miR-let-7c might act as a tumor suppressor, miR-let-7c is highly expressed in invasive tumors compared with that in noninvasive tumors^42^ and can promote the metastasis of cholangiocarcinoma.^39^ Here, for the first time, we explored the roles of miR-let-7c in normal epithelial cells and elucidated the correlation between miR-let-7c and the precancerous transformation of normal epithelial cells.

There are several limitations in our research. First, this study mainly focuses on the roles of secreted sEVs of OSCC but ignores the roles of direct cell contact between cancer cells and adjacent normal cells. Further studies are required to elucidate the possible effects of cellular contact. Second, the lack of a suitable animal model hinders the progress of this research, and greater efforts should be made to resolve this problem in the future.

Regardless, we confirmed that malignant cancer cells can corrupt normal epithelial cells by delivering sEVs, in which miR-let-7c plays a vital role. Our studies link OSCC-derived sEVs to the current understanding of dysplasia and expand their roles in producing long-distance oncogenic effects.

## Materials and methods

### Cell lines and Culture

HIOECs were kindly donated by Professor Cheng-zhang Li and cultured in KGM-gold (Lonza, Switzerland) supplemented with growth factors. The human OSCC cell lines SCC25 and CAL27 were purchased from ATCC (Manassas, US). Ten percent FBS (Gibco, USA) was added to the medium of SCC25 and CAL27 cells. All cells were cultured in a constant temperature and pressure incubator at 37 °C and 5% CO_2_.

### Hematoxylin and eosin (HE) staining

Tumor tissue slides were observed with HE staining according to the conventional protocol.

### Isolation and analysis of small extracellular vesicles

When the cells were cultured to 70%–80% confluence, the supernatant was discarded. The cells were then washed with a phosphate buffer solution (PBS) three times and cultured in FBS-free medium for 24 h. Differential ultracentrifugation was performed to isolate the sEVs in the supernatant using an Optima XE-100 (Beckman Coulter, USA) at 800 × *g* for 10 min, 1 500 × *g* for 15 min, 10 000 × *g* for 35 min, and 110 000 × *g* for 70 min. The sediments were then washed in PBS under the same conditions. sEVs were observed by TEM (HITACHI, Japan). A Nano-ZS ZEN 3600 (Malvern Instruments, UK) was used to measure the hydrodynamic diameter of the sEVs.

### Small extracellular vesicle tracing

To observe the interaction between sEVs and epithelial cells, sEVs were labeled with PKH26 (Sigma–Aldrich, St. Louis, MO). After incubating for 4 h with PKH26-labeled sEVs, HIOECs were observed with a confocal microscope (Olympus, Japan).

### Wound healing and Matrigel invasion assays

The experiments were conducted as described in previous research.^43^ Briefly, the cells were evenly planted on a six-well plate and grown into a confluent monolayer. Subsequently, a scratch was made and marked on the underside of the plate to ensure that the images were observed at identical places at 0 and 48 h. The migrated areas were calculated using Image-Pro Plus 6.0 software.

The Transwell chamber was precoated with 50 μL matrix gel (BD Biosciences, San Jose, CA). A total of 2 × 10^5^ cells were then inoculated into the upper chamber. The invading cells were stained after 48 h and counted for the Matrigel invasion assay. In the above experiments, cell proliferation was inhibited using mitomycin C (Selleckchem, USA).

### Western blot analysis

M-PER (Pierce Inc., USA) was used to extract total protein from the cells and sEVs on ice. Protease inhibitors and phosphatase inhibitors were added to prevent protein degradation. Following ultrasonic vibration for 15 s, the solution was centrifuged at 4 °C for 10 min, and the supernatant was collected. The protein concentration of each sample was measure by a BCA Protein Assay Kit (Thermo Fisher Scientific Inc., USA). The lysate was then separated by 10% sodium dodecyl sulfate–polyacrylamide gel electrophoresis and transferred to a polyvinylidene fluoride membrane. Antibodies were used to incubate at 4 °C overnight after treatment with 5% skimmed milk. The eluted polyvinylidene fluoride membrane was washed three times with Tris-buffered saline-Tween 20, incubated with the corresponding secondary antibody for 1 h, and visualized after adding an enhanced chemiluminescent substrate. Anti-P53, anti-E-cadherin, anti-vimentin, anti-PARP, anti-caspase-3, anti-BAX, and anti-BCL-2 antibodies were obtained from Proteintech (1:1 000; China). Anti-PTEN and anti-N-cadherin antibodies were obtained from Cell Signaling Technology (1:1 000; USA).

### CCK-8 assay

HIOECs were seeded in 96-well culture plates. After incubation with small extracellular vesicles for 24, 48, and 72 h, with no small extracellular vesicles added as a control, cell proliferation was measured using a Cell Counting Kit-8 (Biosharp, China).

### EdU assay

The assay was carried out as previously described.^43^ In summary, HIOECs were uniformly plated into 24-well plates, fixed and stained using the EdU Cell Proliferation Kit (Beyotime, China) as instructed, and finally observed and photographed under a fluorescence microscope (Keyence, Japan).

### 3D culture

For 3D culture, HIOECs were embedded in Matrigel (BD Biosciences, Franklin Lakes, NJ, USA) with 3 000 cells per 30 μL Matrigel/well in 24-well plates. The Matrigel-cell complex was stabilized at 37 °C for 15 min and then added to the medium for culture.

### Immunofluorescence

The cells in plates were fixed with 4% paraformaldehyde for 30 min, treated with 0.2% Triton X-100 (Servicebio, China) for 5 min and blocked with 5% BSA (Servicebio, Wuhan, China) at room temperature for 30 min. Next, the cells were incubated with anti-p53 (1:200; Proteintech, China) and anti-PTEN antibodies (1:200; Proteintech, China) for 24 h and with FITC and Cy3-conjugated secondary IgG (Servicebio, China). Finally, the nuclei were stained with DAPI. The fluorescence microscope (Keyence, Japan) was used to obtain images of the stained cells.

### Cell apoptosis assay

Cells were collected with trypsin, washed with precooled PBS, and then stained using an Annexin V-FITC/PI Apoptosis Kit (MultiSciences, China). Cell apoptosis was analyzed by flow cytometry.

### qRT–PCR

Total RNA of cells and sEVs was extracted using TRIzol reagent (Takara, Tokyo, Japan). cDNA was obtained using miRNA first strand cDNA synthesis (Sangon Biotech, Shanghai, China). After the cDNA was diluted 50 times, the mature miRNA and U6 were quantified with a miRNA qPCR kit (Sangon Biotech, Shanghai, China) and Bio–Rad CFX96 (Bio–Rad Laboratories, USA). The miRNA primers utilized in this research are listed in Table S1.

### Luciferase reporter assay

Luciferase plasmids (pmirGLO; GenePharma Inc, China) encoding the mutant or wild-type 3′ untranslated region (3′UTR) of p53 were treated with hsa-let-7c-5p mimic or NC-mimics and hsa-let-7c-5 inhibitor or NC-inhibitor. Transfection was conducted by Lipofectamine 3000^TM^ (Invitrogen, USA). The Dual-Luciferase Reporter Assay Kit (Promega, USA) was adopted to test luciferase activity after 48 h, as instructed by the manufacturer.

### Statistical analysis

The data were analyzed using GraphPad Prism 6 software (La Jolla, USA) in at least three independent experiments with three replicates, and values are expressed as the means with standard error of mean (SEM). Student’s *t*-test and one‐way analysis of variance were used to analyze data between two groups and multiple groups, respectively. Differences with *P*-values < 0.05 were considered statistically significant (*P < 0.05, **P < 0.01, ***P < 0.001).

## Supplementary information

Supplementary Information
