# Supplementary material for: Cancer cells corrupt normal epithelial cells through miR-let-7c-rich small extracellular vesicle-mediated downregulation of p53/PTEN
Source: Int J Oral Sci. 2022 Jul 19;14:36. doi: 10.1038/s41368-022-00192-2 (PMC9293927; doi:10.1038/s41368-022-00192-2)
Supplement: Supplementary file 1 — Supplementary Information [file 41368_2022_192_MOESM1_ESM.pdf]

Figure S1

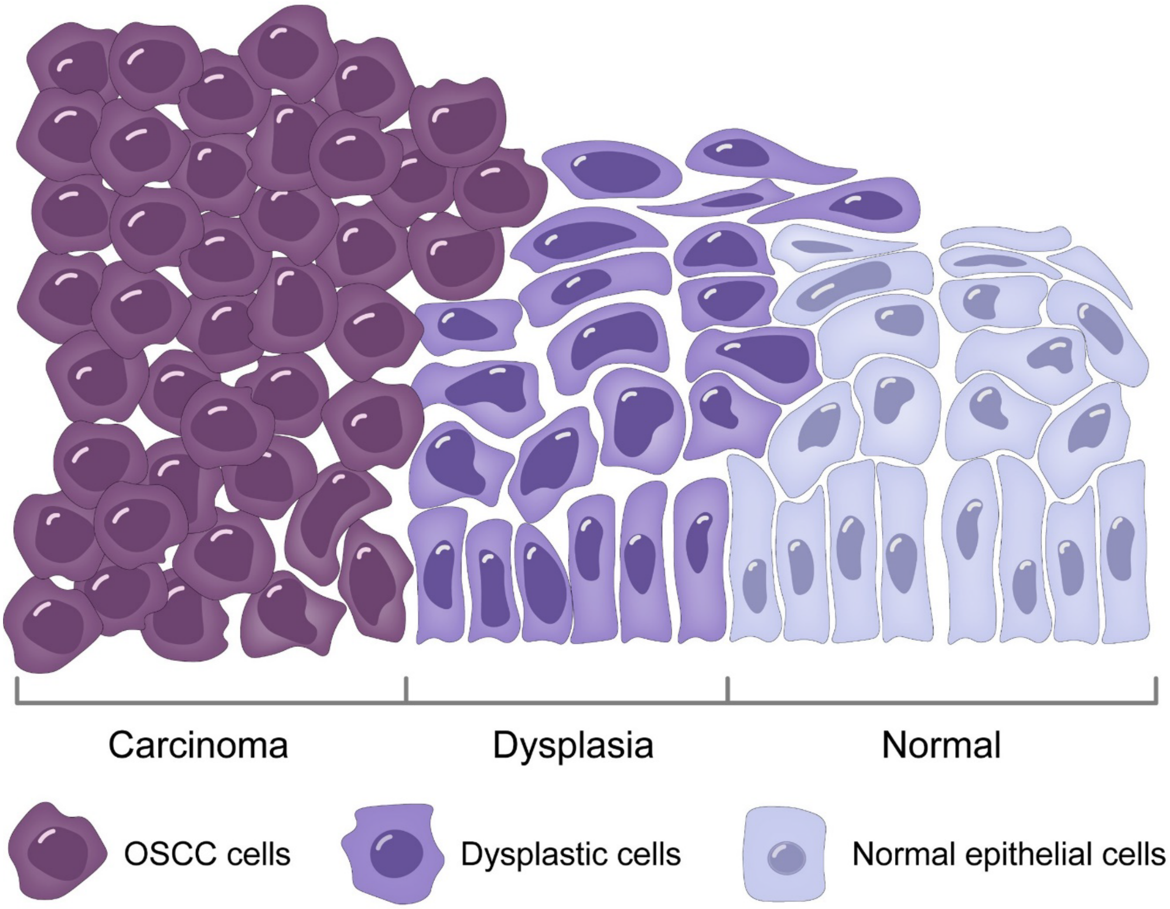

Fig.S1 Progression of OSCC. Pathologically, normal mucous epithelium develops dysplasia followed by carcinoma in situ.

Figure S2

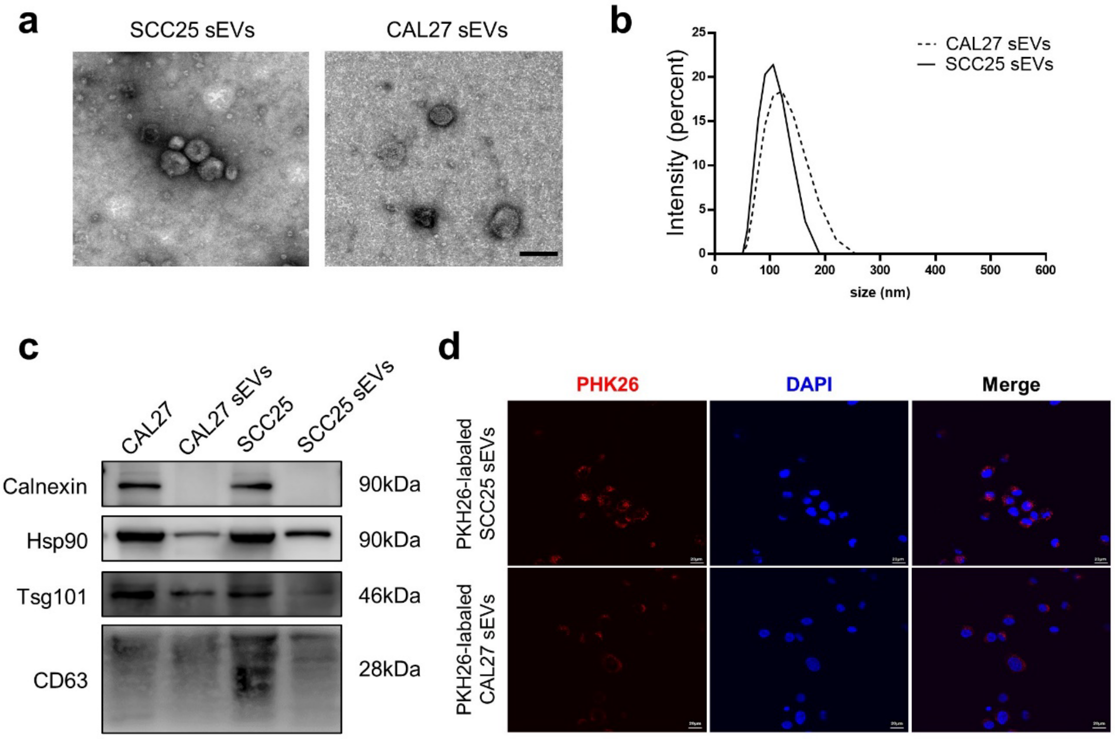

Fig.S2 (**a-b**) SCC25 and CAL27-derived sEVs were identified by TEM and DLS analysis. Scale bar, 100 nm. (**c**) Characterization of sEVs with Western blot. (**d**) Confocal microscope images showed the uptake of PKH26-labeled sEVs in HIOECs. Scale bar, 20 μm. (**e**) Immunofluorescence staining of p53 and PTEN in HIOCEs treated with OSCC-derived sEVs. Scale bars, 20 μm.

Figure S3

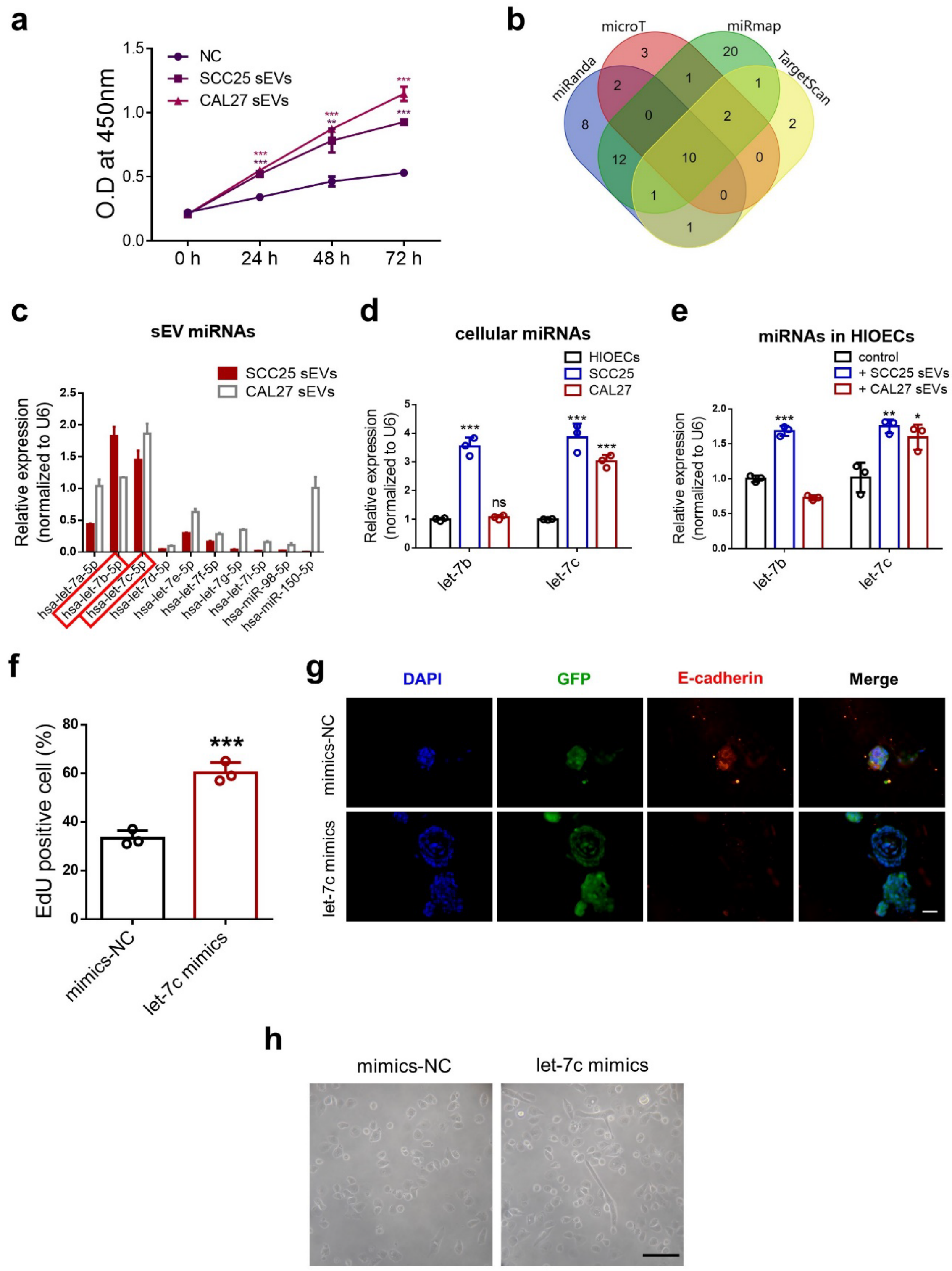

Fig.S3 (**a**) The proliferation ability of HIOECs treated with OSCC-derived sEVs. (**b**) The potential miRNAs that targeting p53. (**c**) The predicted miRNAs in sEVs released by OSCC cells were detected by qRT-PCR. (**d**) The expression level of miRNA let-7b and let-7c in HIOECs, SCC25 and CAL27. (**e**) The expression of let-7b and let-7c in HIOCEs treated with OSCC-derived sEVs. (**f**) The proliferative cells of HIOECs transfected with let-7c mimics. (**g**) Immunofluorescence staining of E-cadherin in HIOECs transfected with let-7c mimics in Matrigel. Scale bars, 100 μm. (**h**) The morphology of HIOECs treated with OSCC-derived sEVs. Scale bar, 50 μm.

Table S1 Sequences of PCR primers used in this study.

microRNA	Targeting sequences (5′ to 3′)
hsa-let-7a-5p	GCGCGTGAGGTAGTAGGTTGTATAGTT
hsa-let-7b-5p	CCGTGAGGTAGTAGGTTGTGTGGTT
hsa-let-7c-5p	CCGCTGAGGTAGTAGGTTGTATGGTT
hsa-let-7d-5p	CCGCAGAGGTAGTAGGTTGCATAGTT
hsa-let-7e-5p	CGCGTGAGGTAGGAGGTTGTATAGTT
hsa-let-7f-5p	CGCGCGCTGAGGTAGTAGATTG
hsa-let-7g-5p	CGCCGTGAGGTAGTAGTTTGTACAGTT
hsa-let-7i-5p	CGCTGAGGTAGTAGTTTGTGCTGTT
hsa-miR-98-5p	GCGCGCTGAGGTAGTAAGTTGTATTG
hsa-miR-150-5p	CTCTCCCAACCCTTGTACCAGTG
hsa-miR-494-5p	CAGGTTGTCCGTGTTGTCTTCTCT
